# Early versus late reversal of diverting loop ileostomy in rectal cancer surgery: a multicentre randomized controlled trial

**DOI:** 10.1038/s41598-023-33006-4

**Published:** 2023-04-10

**Authors:** Mark Bremholm Ellebæk, Sharaf Karim Perdawood, Signe Steenstrup, Sardar Khalaf, Jette Kundal, Sören Möller, Jacob Christian Bang, Jens Støvring, Niels Qvist

**Affiliations:** 1 Research Unit of Surgery, Odense University Hospital, Odense, Denmark; 2 OPEN, Open Patient Data Explorative Network, Odense University Hospital, Odense, Denmark; 3 Department of Clinical Research, University of Southern Denmark, Odense, Denmark; 4 Department of Gastrointestinal Surgery, Slagelse Hospital, Slagelse, Denmark; 5 Department of Surgery, Hospital South West Jutland, Esbjerg, Denmark; 6 Department of Radiology, Svendborg Hospital, Svendborg, Denmark

**Keywords:** Colorectal cancer, Rectal cancer

## Abstract

Diverting loop ileostomy has become routine in low anterior resection (LAR) for rectal cancer. The optimal time for stoma reversal is controversial. The aim of the present study was to compare the results after planned early (within 8–12 days) versus late (> 3 months) stoma reversal. The primary outcomes were morbidity and mortality, as measured by the Comprehensive Complication Index (CCI) within 30 days after stoma reversal, and the secondary outcomes were morbidity and mortality within 90 days after LAR. This was a multicentre trial including all patients scheduled for anterior low resection for rectal cancer with curative intent. Inclusion period was from April 2011 to December 2018. All patients were randomized 1:1 prior to surgery. Among 257 consecutive and eligible patients, a total of 214 patients were randomized: 107 patients to early stoma reversal and 107 to late reversal. A total of 68 patients were excluded for various reasons, and 146 patients completed the study, with 77 in the early reversal group and 69 in the late reversal group. The patients were asked to complete the Gastrointestinal Quality of Life Index before surgery (baseline) and at 6 and 12 months after LAR. Ostomy-related complications were evaluated by dedicated ostomy staff using the validated DET score. ClinicalTrials Identifier: NCT01865071. Fifty-three patients (69%) in the early reversal group and 60 patients (87%) in the late reversal group received the intended treatment. There were no significant differences in CCI within 90 days after index surgery with the LAR and within 30 days after stoma reversal between the two groups. There were no differences in patient-reported quality of life but significantly more stoma-related complications in the late reversal group. A total of 5 patients experienced anastomotic leakage (AL) after stoma reversal, 4 in the early reversal group and one in the late reversal group. Early and late stoma reversal showed similar outcomes in terms of overall complications and quality of life. The risk of developing anastomotic leakage after early ostomy reversal is a concern.

## Introduction

A defunctioning stoma significantly reduces the morbidity of anastomotic leakage (AL) in low anterior resection (LAR) with total mesorectal excision for rectal cancer^1^. A concern may be stoma-related morbidity and complications to stoma reversal, which has been reported with a frequency of 17.3–20%, with major complications ranging from 0 to 7.9% and a mortality rate of 0.4 to 1%^2,3^.

There is no clear evidence for the optimal timing of stoma reversal, and the majority of reports recommend a stoma for approximately 3 months^4–6^. Early reversal has been studied in nine published randomized trials with conflicting results^5,7–14^. The explanation may be different study designs with different periods for stoma reversal, patient selection (benign and malignant conditions or a mixture) and the method of randomization in relation to time from index surgery. A meta-analysis including seven of the trials concluded that early stoma reversal was safe and feasible and associated with a reduced risk of bowel obstruction and a lower rate of stoma-related complications but a higher rate of wound complications^15^. Not surprisingly, another meta-analysis based on 7 of the 9 published studies came to the same conclusion^16^. The first meta-analysis did not include the studies by Elsner et al.^13^ and Gallyamov et al.^14^, and the later meta-analysis did not include the studies by Shah et al.^12^ and Nelson et al.^9^. The study by Elsner et al.^13^ found that early stoma reversal was not associated with a better quality of life up to 4 months after LAR but was afflicted with significantly adverse feasibility and higher morbidity compared with late reversal.

A consistent feature in the method of randomization in the previous studies was a postsurgical randomization, which had a risk of patient selection and did not show the feasibility of early stoma reversal in general. Randomization before the index surgery was only used in one study from 2008, which included a mixture of patients with benign and malignant disease^7^. Given this background, we decided to conduct a study with randomization prior to the index surgery to decrease patient selection as a confounder.

The primary outcomes were morbidity and mortality measured by the Comprehensive Complication Index (CCI)^17^ within 30 days after stoma reversal, and the secondary outcomes were morbidity and mortality (CCI) within 90 days after LAR, ostomy-related complications until stoma reversal and patient-reported quality of life at 6 and 12 months after LAR.

## Materials and methods

### Study design

The trial was designed as a multicentre prospective randomized controlled trial comparing early (8–12 days) versus late (> 3 months) reversal of a diverting loop ileostomy as an intention-to-treat investigation. Patients were recruited from four Danish surgical departments certified for rectal cancer surgery. The average annual volume for rectal resection with primary anastomosis with or without a diverting loop-ileostomy in the 4 centres was a median of 39 (range 11–83) during the inclusion period from April 2011 to December 2018 and with the last follow-up in December 2019. Screening for eligibility, inclusion, and randomization was performed at the outpatient clinic before surgery.

### Important changes to methods after trial commencement

The trial period was prolonged by 2 years, as the inclusion rate was slower than anticipated and due to different times of start-up in the participating centres. The initial power calculation was based on the assumption of a 5% complication rate in the early stoma reversal group and 30% in the late stoma reversal group. With a power of 80% and a 5% significance level, 75 patients in each group would be required (nQuery Advisor 6.01; GraphPad Software DBA Statistical Solutions, San Diego, USA). As we expected that at least 25% would have to be excluded because of operative deviation from the planned LAR with a diverting ileostomy or other reasons, at least 200 patients were needed.

An interim analysis (01.11.2015) after inclusion of half of the patients showed no differences in the complication rates between the two groups and considerably lower than the 30%. By continuing the study by registration of the complication rate, only, a type 2 error in the interpretation of the results might be a risk. Therefore, CCI was considered more appropriate to use. With the initial intention to include 150 patients our power calculation showed that a difference in CCI on 5 between the two groups would be significant. This was considered clinically relevant, as we would go for only a small difference in the argument to change from a late to an early stoma reversal in the clinical setting.

### Participants

Patients had to be at least 18 years old and have a histologically proven adenocarcinoma of the rectum at or below 15 cm from the anal verge. All patients underwent a preoperative CT scan of the chest and abdomen, pelvic MRI, and endorectal ultrasound. All patients were preoperatively evaluated at a multidisciplinary team (MDT) conference. Only patients scheduled for LAR with curative intention were considered eligible.

### Intervention

LAR was performed with the principles for total mesorectal excision (TME). All anastomoses were stapled and tested intraoperatively for leakage. Pelvic drainage was performed at the discretion of the surgeon. Enhanced Recovery After Surgery (ERAS) principles were not standardized in the centres.

On postoperative Day 7, all patients were scheduled for a CT scan of the abdomen with intravenous contrast and a water-soluble rectal enema. Patients randomized and scheduled for early stoma reversal also underwent preoperative endoscopy. Early stoma reversal was cancelled in case of suspicion of an AL at endoscopy and/or CT scan and in the case of other complications or conditions considered absolute or relative contraindication for early stoma reversal. The ileostomy reversal technique was not standardized and left to the discretion of the surgeon.

### Outcomes

Complications were measured by the CCI^17^. The CCI is based on grading by the Clavien‒Dindo Classification and includes every complication that occurs after an intervention^18^. The overall morbidity is reflected on a scale from 0 (no complication) to 100 (death).

Ostomy-related complications were evaluated by dedicated ostomy staff using the validated DET score^19^ assessing the severity and extent of peristomal skin damage in three areas—discolouration (D), erosion (E) and tissue overgrowth (T)—at 3, 10, 17, 30, 60, 90, 180, and 240 days after stoma formation or until stoma reversal. The Gastrointestinal Quality of Life Index (GIQLI) questionnaire^20^ was completed before surgery (baseline) and at 6 and 12 months after LAR.

The following preoperative data were collected prospectively: sex, age, body mass index (BMI), smoking habits, alcohol consumption, preoperative chemoradiation (CRT), and tumour height from the anal verge. Physical status was assessed by the American Society of Anesthesiologists (ASA) classification and World Health Organization (WHO) performance status.

Perioperative data collected were the type of surgery (open, laparoscopic, robot-assisted, or laparoscopic transanal total mesorectal excision), conversion to laparotomy, anastomotic height above the dentate line, number of cross staples, blood loss and placement of the pelvic drain. Postoperative data on the TNM stage were obtained from the histology report. Any complications within 90 days after LAR and within 30 days after stoma reversal were prospectively registered according to the Clavien‒Dindo (CD) classification^18^ and summarized at the individual level by the CCI^17^. Any anastomotic leakage was also categorized according to ISREC classification^21^. Data collected at ostomy reversal were the duration of surgery, intestinal resection, and conversion to laparotomy.

All data were entered prospectively into a Research Electronic Data Capture (REDCap) database hosted by the Open Patient Data Explorative Network (OPEN). Surgical complications were registered by the surgeons, and stoma-related complications according to the DET were registered by the stoma nurses.

### Randomization

Patients were randomized either to the intervention group with early ostomy reversal (intended 8–12 days after stoma creation) or to the control group with late reversal (> 3 months). Randomization was a computer-generated central 1:1 randomization and was performed before the index LAR operation. Blinding of the intervention for the patient and surgeons was not possible.

### Statistical analysis

Univariate analyses were performed on the individual complication types (intestinal obstruction, AL, etc.) and on the overall complication rate. Fisher's exact or chi-squared test was used to compare the treatments, depending on the number of observations. Complications as a whole (CCI) were compared between treatments by linear regression with bootstrapped standard errors.

Patient characteristics were summarized as frequencies and proportions (for categorical variables), as the mean values  ± standard deviations for normally distributed continuous variables and as medians with quartiles and minimum and maximum values for nonnormally distributed continuous variables. Categorical characteristics were compared using Fisher's exact test, and continuous characteristics were compared with the Wilcoxon rank-sum test. The DET scores and GIQLI questionnaire were compared by mixed-effects linear regression, considering the repeated measurements from the same patient by a random intercept. A *P* value < 0.05 was considered statistically significant. Statistical calculations were performed using Stata software (StataCorp 2017. Stata Statistical Software: Release 15. College Station, TX: StataCorp LLC).

### Ethics and approvals

This study was performed in line with the principles of the Declaration of Helsinki. Approval was granted from the Regional Scientific Ethical Committees of Southern Denmark (ID: S-20110026) and the Danish Data Protection Agency (2008-58-0035). The ClinicalTrails.gov Identifier is NCT01865071 (First Posted 30.05.2013). Written informed consent was obtained from all patients.

## Results

**Figure 1:**
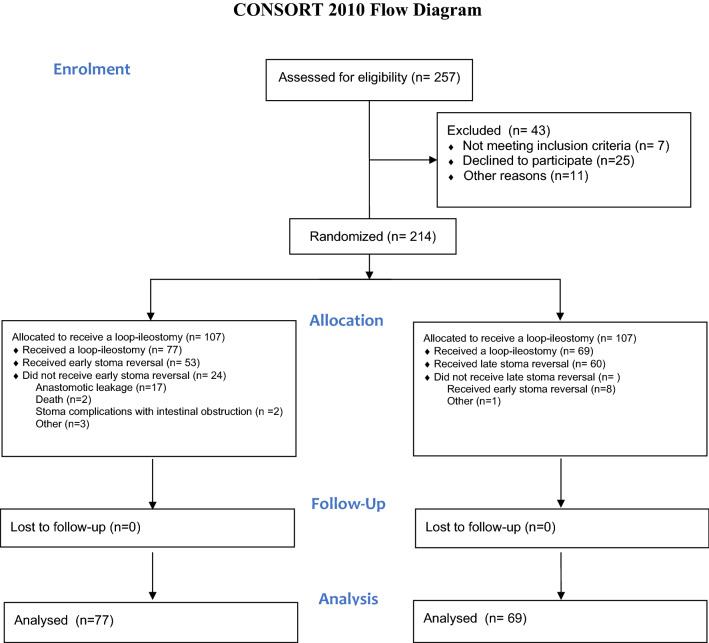
CONSORT flow diagram for enrolment, allocation and follow-up.

Among a total of 257 eligible and consecutive patients, 214 patients were randomized: 107 patients to early stoma reversal and 107 to late reversal. A total of 68 patients were excluded: abdomino-perienal rectum excision (29), no stoma formation (28), withdrawal of consent (3), Hartmann’s procedure (2) and others (6). Thus, a total of 77 patients in the early reversal group and 69 patients in the late reversal group completed the study (Fig. 1). Baseline demographics and surgical and clinicopathologic characteristics were comparable between the two groups (Table 1).

**Table 1:** Demographic and clinical characteristics of the included patients who underwent early or late reversal of a diverting loop ileostomy after low anterior resection.

Characteristics	Randomized to early stoma reversal N = 77	Randomized to late stoma reversal N = 69
Sex		
Male	53 (69%)	52 (75%)
Female	24 (31%)	17 (25%)
Age (years, mean and SD)	66.3 (8.1)	64.5 (9.1)
BMI (mean and SD)	26.0 (3.71)	27.2 (5.71)
ASA score		
1	28 (36%)	27 (39%)
2	43 (56%)	36 (52%)
3	6 (8%)	6 (9%)
WHO performance status (PS)		
PS 0	62 (81%)	60 (90%)
PS 1	10 (13%)	5 (7%)
PS 2	2 (3%)	0 (0%)
Unknown	3 (4%)	3 (3%)
Smoker		
No	59 (77%)	53 (79%)
Yes	15 (19%)	12 (18%)
Unknown	3 (4%)	2 (3%)
Alcohol abuse		
No	61 (79%)	61 (88%)
Yes	11 (14%)	6 (9%)
Unknown	5 (6%)	2 (3%)
Cardiopulmonary disease		
No	32 (42%)	37 (54%)
Yes	43 (56%)	31 (45%)
Unknown	2 (3%)	1 (1%)
Diabetes		
No	63 (82%)	60 (87%)
Yes	10 (13%)	8 (12%)
Unknown	4 (5%)	1 (1%)
Neurological disease		
No	67 (87%)	65 (94%)
Yes	6 (8%)	3 (4%)
Unknown	4 (5%)	1 (1%)
Tumour characteristics and neoadjuvant therapy		
pT stage		
T0	5 (6%)	4 (6%)
T1	5 (6%)	2 (3%)
T2	17 (22%)	25 (36%)
T3	48 (62%)	37 (54%)
T4	2 (3%)	1 (1%)
pN stage		
N0	38 (49%)	34 (49%)
N1	23 (30%)	19 (28%)
N2	14 (18%)	14 (20%)
Nx	2 (3%)	2 (3%)
cM stage		
M0	68 (89%)	64 (94%)
M1	8 (11%)	4 (6%)
Tumour height from anal verge (cm, mean and SD)	8.93 (2.22)	8.97 (2.49)
Neoadjuvant treatment		
None	56 (73%)	53 (77%)
Radiation	2 (3%)	2 (3%)
Radiation chemotherapy	12 (16%)	9 (13%)
Chemotherapy	7 (9%)	5 (7%)
Surgical data from index operation		
Operation time (min, mean and SD)	274 (71)	257 (70)
Bleeding (ml, mean and SD)	163 (203)	142 (147)
Anastomotic height from anal verge (cm, mean and SD)	4.55 (1.01)	4.64 (0.96)
Drain		
No	40 (52%)	37 (54%)
Yes	32 (42%)	28 (41%)
Unknown	5 (6%)	3 (4%)
Number of cross stapling		
1	12 (16%)	10 (15%)
2	29 (38%)	22 (32%)
3	12 (16%)	14 (21%)
4	2 (3%)	1 (1%)
5	1 (1%)	0 (0%)
Unknown	21 (27%)	21 (31%)
Type of operation		
Open	1 (1%)	0 (0%)
Laparoscopic	36 (47%)	39 (57%)
converted	4 (5%)	2 (3%)
Robot assisted	27 (35%)	19 (27%)
converted	1 (1%)	3 (4%)
Ta-TME	8 (11%)	6 (9%)

BMI, Body mass index; ASA, American Society of Anesthesiologists; pT, Pathological tumour stage; pN, Pathological lymph node stage; cM, Clinical metastatic stage; Ta-TME, Transanal total mesorectal excision.

*Maximal recommended limits of alcohol intake (units per week): males = 14 and females = 7 (Danish Health Authorities).

In the group randomized to early stoma reversal, only 53 patients (69%) received the intended treatment. Stoma reversal was postponed by 3 months in 20 patients due to AL (17 patients), stoma complications with intestinal obstruction (2 patients), and other reasons (1 patient). Four patients did not have stoma reversal. Of these, two died on postoperative Days 6 and 12 due to complications of AL, and another 2 ended up with diverting loop ileostomy as a permanent stoma for other reasons.

In the late reversal group, 60 patients (87%) received the intended treatment. Eight patients underwent earlier reversal due to ileus in seven and for an unknown reason in one. One patient ended up with diverting loop ileostomy as a permanent stoma**.**

The total CCI (surgical + medical) within 30 days after stoma reversal in the early and late reversal groups was 7.58 and 6.66, respectively (*P* = 0.716). The values for the 90-day CCI after LAR were 23.1 and 19.05 (*P* = 0.361). The severity and type of complications appear in Table 2. A total of 5 patients experienced AL after stoma reversal at the colorectal anastomosis (all CD grade 3b), with four in the group randomized to early reversal and one randomized to late reversal. In all 5 cases, the loop ileostomy stoma was reinserted.

**Table 2:** Severity and type of postoperative complications in the two groups. A patient may have more than one complication.

Within 90 days after low anterior resection (LAR)	Randomized to early reversal N = 77	Randomized to late reversal N = 69
Clavien‒Dindo-classification (worst)	N (%)	N (%)
1	7 (9%)	5 (7%)
2	8 (10%)	4 (6%)
3a	8 (10%)	5 (7%)
3b	15 (19%)	17 (25%)
4a	5 (6%)	2 (3%)
4b	0 (0%)	0 (0%)
5	2 (3%)	0 (0%)
Type of complication		
Bleeding	4 (5%)	1 (1%)
Fascial dehiscence	0 (0%)	0 (0%)
Ileus	20 (26%)	21 (30%)
Wound abscess	0 (0%)	2 (3%)
Intraabdominal abscess	8 (10%)	6 (9%)
Stoma complications	13 (17%)	14 (20%)
Leak (Clavien‒Dindo-classification)	28 (36%)	14 (20%)
< 3b	12	5
> 3b	16	9
Leak (According to ISREC)		
A	9	2
B	7	8
C	12	4
		1
Other	3 (4%)	3 (4%)
Within 30 days after stoma reversal		
Clavien‒Dindo-classification (worst)		
1	2 (3%)	0 (0%)
2	6 (8%)	3 (4%)
3a	0 (0%)	4 (6%)
3b	6 (8%)	4 (6%)
4a	0 (0%)	1 (1%)
Type of complication		
Bleeding	4 (5%)	0 (0%)
Fascial dehiscence	3 (4%)	1 (1%)
Ileus	6 (8%)	2 (3%)
Leakage (Colorectal anastomosis)	4 (5%)	1 (1%)
(Clavien‒Dindo-classification)		
< 3b	0	0
> 3b	4	1
Leak (According to ISREC)		
A	0	0
B	0	0
C	4	1
Other	4 (5%)	4 (6%)

The mean time from LAR to stoma reversal was 47 and 154 days in the early and late reversal groups, respectively (*P* < 0.001). In the stoma reversal procedure, there was no significant difference in the duration of surgery, amount of bleeding, frequency of intestinal resection or laparotomy between the two groups (Table 3).

**Table 3:** Fate of the loop ileostomy, time to stoma reversal and surgical procedure at reversal in the two groups.

Fate of ileostomy	Early reversal group	Late reversal group
Never closed	4	1
Closed	73	68
Time with ileostomy (days, mean and 95% CI) €	47 (30–63)	154 (130–178)
Operation time (min, mean and 95% CI)	69 (61–77)	63 (57–69)
Bleeding (ml, mean and 95% CI)	52 (21–83)	31 (17–46)
Intestinal resection		
No	32 (44%)	32 (47%)
Yes	39 (54%)	35 (52%)
Unknown	2 (1%)	1 (1%)
Laparotomy		
Yes	10 (14%)	6 (9%)
No	61 (84%)	62 (91%)
Unknown	2 (3%)	0 (0%)

€, missing data; 4 in the early group and 1 in the late group; CI, 95% Confidence Interval.

The overall mean DET score was higher in the late reversal group (0.63; 95% CI 0.08, 1.17; *P* = 0.024).

No significant difference in quality of life was found at the 6- and 12-month follow-ups after LAR (Table 4).

**Table 4:** Quality of life at baseline and at 6 and 12 months after low anterior resection.

Time point	Early reversal N = 77		Late reversal N = 69	
	Completed	Mean (SD)	Completed	Mean (SD)
Baseline	50	107.06 (13.65)	44	105.23 (12.86)
6 months	54	106.07 (13.44)	46	101.00 (14.12)
12 months	53	107.09 (14.34)	53	105.21 (10.95)

SD, standard deviation.

## Discussion

The present study showed no significant difference in postoperative complications after early or late reversal of a diverting loop ileostomy as assessed by CCI within 30 days after stoma reversal and within 90 days after the LAR. There were no differences in quality of life at the 6- or 12-month follow-up. Most importantly, a total of 5 patients developed clinical anastomotic leakage after stoma reversal, with four randomized to early reversal and one randomized to late reversal. The patient who developed AL in the late reversal group underwent early reversal due to stoma complications. In all 5 patients, postoperative CT scanning did not reveal any suspicion of AL, which may indicate that early stoma reversal may induce AL.

The study also showed that early reversal was feasible in 69% of patients only and that more than 10% randomized to late reversal underwent an earlier reversal primary due to stoma-related complications.

The major reason for not undergoing early reversal was anastomotic leakage demonstrated in 17 patients (22%) at routine postoperative CT scanning and/or endoscopy. Four of these patients developed overt clinical symptoms that needed intervention, and two of them died. In the remaining 13 patients, the leakage was subclinical.

Seven of the nine previous RCTs showed that early closure was feasible, with similar complication profiles in patients randomized after index surgery and without clinical, radiological, or endoscopic signs of AL^7–12,14^. Significantly fewer postoperative complications at early reversal have been reported in RCTs^8^, but two other studies were terminated prematurely due to an increased risk of complications at early reversal^5,13^. The predefined time to early closure varied from 8 to 30 days^7,8,10,11^. Eight of the nine RCT studies used randomization after the index procedure with inclusion of patients with an uneventful recovery, in good general condition, and with normal rectal enema/computed tomography (CT) and/or endoscopy^5,8–12^. Two studies included both acute and elective cases with different diseases and surgical procedures^9,12^. One study included a mixture of patients with benign and malignant disease, with randomization before the index surgery^7^.

Theoretically, there may be several advantages to the early reversal of a diverting ileostomy, as it may relieve ostomy-related complications. This was also demonstrated in our study, as some of the patients in the late reversal group underwent early reversal due to stoma complications. Although stoma-related complications may seem less severe than other surgical and medical complications, they can be bothersome, distressing, and embarrassing for the patient. In the study by Danielsen et al.^8^, stoma-related complications were reported in 24% of the patients in the early reversal group compared to 75% in the late reversal group. In a later publication with the same patient material, they found no differences in quality of life between early and late reversal measured by the SF36 and EORTC QLQ-C30 and QLQ-CR29 questionnaires. Another important issue may be health care costs in relation to ostomy products and outpatient controls^10,22^, but this was not an aim of our study.

The development of AL after early reversal in a significant number of patients in our study is a concern, and it is problematic that neither CT scan nor endoscopy was able to demonstrate any leakage in the patient. This might be true, although it is hard to believe that an early reversal on postoperative Days 8–12 may cause anastomotic dehiscence. Another problem in early versus late reversal might be the logistical problems of planning the “subacute” stoma reversal 8 days after the initial surgery.

### Strengths and limitations

The present design with randomization prior to surgery is a strength because it reduces the risk of patient selection and outlines the problems and shortcomings in early stoma reversal in surgery for rectal cancer. Another strength is the intention-to-treat analysis. No blinding of the randomization for patients, surgeons or medical staff at index surgery has an inherent bias with respect to decisions on the choice and preference of surgical procedure.

An obvious limitation was the long inclusion period with the risk of changes in clinical practice over time. Including only four centres is a strength, which ensures a more homogeneous treatment of patients throughout the study period. The different surgical approaches (robotic, laparoscopic and transanal total mesorectal excision) are not considered a limitation because none of them has proven to be superior or inferior with respect to postoperative complications^23,24^ or rates of conversions^25^.

An important limitation might be the registration of postoperative complications. In those patients in the late reversal group who underwent early reversal for various reasons, the postoperative complications of stoma reversal were included in the 90-day complications. Stoma reversal occurred in only a minority of patients, with few minor complications. This might explain the trend with a higher CCI in the late reversal group compared to the early reversal group, but this difference might also be due to stoma-related complications.

Another important limitation or consideration might be the change in the primary outcome form complicate rates to CCI. The design of the study remained otherwise unchanged, and the change probably decreased the risk of a type 2 error.

## Conclusions

The present study showed that the CCI score was similar after early or late reversal of a diverting ileostomy with LAR for rectal cancer. A concern was the risk of developing anastomotic leakage after early reversal.

## Data Availability

Anonymized raw data can be provided on request from the first author by email.
